# WHO malaria nucleic acid amplification test external quality assessment scheme: results of eleven distributions over 6 years

**DOI:** 10.1186/s12936-025-05282-0

**Published:** 2025-03-23

**Authors:** Rebecca M. Thomson, Jane A. Cunningham, Michelle M. Gatton, Sean C. Murphy, Maria de la Paz Ade, Xavier C. Ding, Sandra Incardona, Eric Legrand, Naomi Lucchi, Didier Menard, Samuel L. Nsobya, Agatha C. Saez, Jaya Shrivastava, Peter L. Chiodini

**Affiliations:** 1 London, UK; 2 World Health Organization, Geneva, Switzerland; 3 Centre for Immunology and Infection Control, Faculty of Health, Queensland University of Technology, Brisbane, Australia; 4 Malaria Molecular Diagnostic Laboratory, Laboratory Medicine & Pathology, University of Washington, Seattle, USA; 5 Seattle Malaria Clinical Trials Center, Fred Hutchinson Cancer Research Center, Seattle, USA; 6 Department of Communicable Diseases and Health Analysis, Pan American Health Organization, World Health Organization, Washington, DC USA; 7 Abbot Rapid Diagnostics, Baar, Switzerland; 8 MCD Global Health, Paris, France; 9 Malaria Biology and Vaccine Unit, Parasite and Insect Vector Department, Institut Pasteur, Paris, France; 10 Malaria Branch, Division of Parasitic Diseases and Malaria, Center for Global Health, Centers for Disease Control and Prevention, Atlanta, USA; 11 Malaria Genetics and Resistance Team (MEGATEAM), UR 3073-Pathogens Host Arthropods Vectors Interactions, Université de Strasbourg, Strasbourg, France; 12 Department of Pathology, School of Biomedical Science, Makerere University Uganda, Kampala, Uganda; 13 UK NEQAS Parasitology, UK Health Security Agency, London, UK; 14 The Hospital for Tropical Diseases, London, UK

## Abstract

**Background:**

The World Health Organization (WHO) recommends parasite-based diagnosis of malaria before treatment. The use of nucleic-acid amplification (NAAT) for detection of *Plasmodium* spp. has expanded rapidly in recent years, for epidemiological research globally and clinical care in high-resource settings. Data from NAATs are frequently used to inform policy decisions, so quality control is essential to ensure results are reliable and comparable. Therefore, robust quality control, including an external quality assessment (EQA) scheme targeting malaria NAATs, is essential. The WHO Global Malaria Programme and the UK National External Quality Assessment Service (UK NEQAS) have collaborated since 2017 to implement a global malaria NAAT EQA scheme.

**Methods:**

Panels of specimens containing five major species of human-infecting *Plasmodium* at various parasite concentrations and negative samples were created in lyophilized blood (LB) and dried blood spot (DBS) formats. Two distributions per year were sent, containing five LB and five DBS specimens. Samples were validated by expert referee laboratories prior to distribution. Between 37 and 51 laboratories participated in each distribution and submitted results online. Participants were scored based on their laboratory's stated capacity to identify *Plasmodium* species, and individual laboratory reports were sent which included performance comparison with anonymized peers. Change in performance over time was calculated using a generalized mixed model with a logit link function.

**Results:**

Participating laboratories were located in 42 countries. Sample format (DBS or LB) and parasite density were found to significantly affect performance, while referee labs performed better at identifying *P. falciparum* samples than non-referee labs. Performance of laboratories improved significantly over time, especially for lower density and *P. falciparum* samples.

**Conclusions:**

Results from the first eleven distributions indicate that the EQA scheme has facilitated improved performance of laboratories over time, highlighting the value of implementing such programmes. EQA schemes are critical to safeguarding the reliability of data and diagnoses, especially in situations where NAAT methodologies and protocols are used. In future, funders should make participation in an EQA scheme a requirement for laboratories, and countries can take initiatives to embed such schemes into their own national assessment programmes.

**Supplementary Information:**

The online version contains supplementary material available at 10.1186/s12936-025-05282-0.

## Background

Malaria remains a significant challenge to global health, with 608,000 deaths from the disease in 2022, the majority occurring in sub-Saharan Africa [1]. Accurate and timely diagnosis is crucial for effective case-management of *Plasmodium* infection and elimination efforts in malaria-endemic countries, and since 2010 the World Health Organization (WHO) has recommended confirmation with parasite-based diagnosis of malaria before treatment is given [2]. The main tools for diagnosing malaria in endemic countries are microscopy and antigen-detecting malaria rapid diagnostic tests (RDTs).

The use of nucleic acid amplification-based tests (NAATs) for detection of *Plasmodium* infection has expanded significantly in recent decades. Since the identification of *Plasmodium* 18S ribosomal RNA [3] and the development of the first polymerase chain reaction (PCR) for *Plasmodium* by Snounou and colleagues in 1993 [4], numerous more advanced and/or modified NAAT methods have been developed, such as nested, multiplex and real-time PCR, reverse-transcriptase PCR, loop-mediated isothermal amplification, nucleic acid sequence-based amplification assays and cluster regularly interspaced short palindromic repeats (CRISPR)-based detection [5–11]. NAATs are commonly used in malaria research studies such as clinical trials for new anti-malarial medicines or vaccines, drug efficacy studies, as a reference standard for evaluations of new diagnostics, or for epidemiological studies [12, 13]. Their use for diagnosis in high-income countries is also increasing. NAATs have a lower limit of detection than other assays (down to as few as 1–100 parasites/mL of whole blood in some cases) [8, 14–16].

Due to their very high sensitivity, NAATs are attractive tools to consider for use in low transmission countries moving towards malaria elimination, where the majority of infections are of very low parasite density and often below the limit of detection of RDTs and microscopy [17]. If *Plasmodium falciparum* and *Plasmodium vivax* are both endemic, but largely undetectable by non-NAAT methods, NAATs could be used to identify and enable appropriate treatment in elimination efforts such as mass screen and treat programmes [18].

As well as detecting the presence of *Plasmodium* and species identification, NAATs have several other uses such as to study presence of molecular markers including those conferring resistance to anti-malarials, or to assess whether *P. falciparum* parasites have deletions in their *pfhrp2/3* genes, which can lead to erroneous results on the most commonly-used RDTs [19]. NAATs allow for detection of mixed or multiclonal infections, whereas RDTs often cannot detect mixed infections and both they and microscopy are not capable of detecting multiclonal infections. Whole genome sequencing can be used to conduct diversity studies, for example of surface proteins, and can be used to discover medically important variants such as single nucleotide polymorphisms [20], or to distinguish between recrudescence and reinfection [21, 22]. Reliable and accurate NAAT assays are needed to support assessments of efficacy of new malaria vaccines in development and in the early stages of use; of new therapeutics and to assess accuracy of new diagnostics; along with supporting epidemiological understanding of malaria and informing how and where to strengthen surveillance efforts. Thus, data from NAATs are frequently used to inform policy decisions for vaccines, medicines and diagnostics, so quality control is essential to ensure the results generated by NAATs are reliable and also comparable between the laboratories conducting them. Quality assessment schemes can facilitate improvement in laboratory performance and in harmonizing methodologies [23]. While other diagnostic techniques, such as microscopy and RDTs, have their own versions of quality assurance schemes [24, 25], EQA schemes for malaria NAATs are rare.

The WHO Global Malaria Programme and the UK National External Quality Assessment Service (UK NEQAS) Parasitology collaborated to launch the WHO Malaria NAAT EQA scheme in January 2017, targeting laboratories in low and high malaria transmission and resource settings to promote reliability and comparability of molecular data. Panels of *Plasmodium* samples are shipped to participating laboratories twice a year, and after analysis and submission of results the laboratories can assess their performance over time and in relation to other participating laboratories, although all results by laboratory remain confidential.

Results of the first three distributions of this scheme were published by the same authors in 2020 [26]. Those results showed that the type of sample [dried blood spots (DBS) or lyophilized blood (LB)], *Plasmodium* species, and being a referee laboratory were significant factors in performance. As the scheme has progressed, the additional data obtained have allowed for more in-depth analysis of changes in performance of laboratories over time and other factors affecting performance. This paper presents data from the first eleven distributions of the scheme along with challenges faced by laboratories and scheme coordinators.

## Methods

### Enrolment of laboratories

In late 2014, reference and research laboratories in all WHO regions were approached and asked to complete a survey regarding malaria NAAT activities and invited to join the scheme. In order to identify as many eligible laboratories as possible, laboratory networks supporting clinical trials and malaria activities were approached. Awareness of the scheme is continually being increased through a range of mechanisms such as presentations at international conferences and meetings, and creation and maintenance of a WHO webpage featuring relevant documentation and contact information. Donors funding research that is reliant on molecular methods are encouraged to provide the budget and require participation in the scheme. As such, laboratories have continued to join the scheme at every distribution since the beginning.

For the first six distributions, participation was free. In 2020, a tiered fee system was introduced, based on the location (high/ low-income country) and type of laboratory (private, reference, or research laboratory).

### EQA source material

Details of the source materials and preparation of the EQA samples have been published previously [26]. Briefly, leftover clinical samples consisting of EDTA-anti-coagulated peripheral blood were diluted in whole blood regarded as *Plasmodium* negative by UK NHS Blood and Transplant (UKNHSBT), within 48 h of receipt from the diagnostic laboratory. Pre-dilution parasite densities were determined by expert microscopists from UK NEQAS Parasitology, counting the number of parasitized cells in a sample size of 10,000 red blood cells on thin blood films to obtain a percentage parasitaemia and converted to parasite density (number of parasites per µL) using the red cell count. For samples included in the first distribution, red cell counts of 5 × 10^12^ per litre was assumed, while red cell counts in subsequent distributions were determined in the initial, pre-dilution samples using a C-Chip DHC-N01 Disposable haemocytometer (NanoEnTek Inc. via MT Promedt Consulting GmbH, Germany). For cultured parasites, a thin blood film was made from the undiluted culture and a haemocytometer was used from the outset to count erythrocytes in all cases, in order to obtain the pre-dilution parasite density in parasites per µL of synchronized ring-stage parasites. For all samples, clinical and cultured, dilution to the desired parasite density was performed using parasite-negative whole blood supplied by UK NHSBT. All positive and negative samples were confirmed by PCR at multiple referee laboratories after sample production.

### Panel composition

Each panel consisted of *Plasmodium* positive and negative samples and included five DBS samples, containing 50 µL of blood per spot, and five LB samples, containing 500 µL of blood per vial. Concentration of samples ranged from 0.018 to 1,100,000 parasites/µL (Table 1). Not every species was included in each distribution.

**Table 1.** Characteristics of distributions 1–11 EQA panels shipped to participants

	Total No. of dried blood spots	Total No. of lyophilised blood samples	Concentration range (parasites/µL)
Negative	14	12	–
*P. falciparum*	14	16	0.05–1,100,000
*P. vivax*	8	11	0.018–400
*P. knowlesi*	3	6	1–2000
*P. malariae*	9	5	2.6–800
*P. ovale*	7	5	10–280^a^

^a^Three *P. ovale* samples were pooled samples and had unspecified concentrations

Paired samples were included in six distributions. These samples had identical species and concentrations, in DBS and lyophilized blood forms, to enable direct comparison of performance between these two formats. Eleven pairs were included, totalling 22 samples.

### Panel distribution

The first panel of samples was shipped in January 2017. Panels were shipped roughly every six months, with the 11th distribution being sent in September 2022. Most laboratories received samples sent at ambient temperature via courier. In some cases, samples were sent via air freight to the nearest airport for collection.

### Referee laboratories

Seven laboratories were selected to be referee laboratories, based on their experience and publication record of using a range of NAATs and location of their laboratory [27]. These labs conducted molecular analysis on samples prior to distribution to the wider group of participating labs, for confirmation of parasite content and concentration. Referee labs were blinded to the intended results during their referee role as well as during the later panel testing phase. Referee laboratories were unaware in which future distributions the samples would be included, and different sample IDs were used for the EQA scheme distributions. Six of these laboratories also participate in the EQA scheme.

### Data reporting and analysis

After each shipment of samples, a web portal hosted by UK NEQAS was open for six to eight weeks for participants to submit their results. Within 24 h of closing of each distribution, intended results were uploaded on the participant’s portal stating the actual contents of each sample. A detailed report stating the performance of the individual laboratories in that distribution and their scores was uploaded within four to six weeks of closing of each distribution.

Before enrolling into the scheme, laboratories were asked to provide information on what plasmodia they were able to detect to species and genus level, which were then compiled to create an individual laboratory profile. Apart from the initial results, results presented in this paper are based on results adjusted according to the laboratory profiles.

Data analysis apart from that based on submission number was performed using Stata15 (Stata Corp, College Station, USA). Differences in performance were assessed using design-based F-test.

### Analysis of change of performance over time based on submission number

Since laboratories could join the scheme at any time, a new variable was created which indicated the submission number for each laboratory/submission combination. Laboratories which joined the scheme at the eleventh distribution will only have data for one submission (on distribution eleven), while laboratories that joined at the start of the scheme and participated in every distribution will have data for submission numbers 1 to 11. As an example, if a laboratory joined the scheme at the beginning but missed three distributions and had submitted results in eight distributions by distribution eleven, their highest submission number would be eight.

Fewer *Plasmodium knowlesi*, *Plasmodium malariae* and *Plasmodium ovale* samples had been included in the scheme by distribution eleven than the number of *P. falciparum* or *P. vivax* samples. Therefore, these three species have been combined into one group and analysed together to ensure sufficient data for modelling. Additionally, not every species, sample type and density combination were included in each distribution. Laboratory submission numbers cannot exceed the last distribution at which a particular species/sample format/density combination was used. For instance, *P. falciparum* DBS samples with < 100 parasites/µL were only included in distributions 1 and 4, meaning laboratory performance data can only have submission numbers 1 to 4 for this combination. The range of submission numbers varied from 3 to 11, dependent on the species/sample format/density combination. A density threshold of 100 parasites/µL was used for this analysis, as opposed to 2 parasites/µL used for the previous analysis, as there were too few samples of below 2 parasites/µL to enable robust modelling.

The change in performance in terms of correctly identifying a sample within a laboratory was assessed using a generalized mixed model with a logit link function, laboratory as the cluster variable and submission number as the independent predictor. To utilize all the data, but not extrapolate beyond the available data, separate models were created for each species/sample format/density combination. Only combinations used at least once after the third distribution are modelled (i.e. the range of submission numbers must exceed three). Analysis was conducted using the GAMLj module in Jamovi (Gallucci, M. (2019). *GAMLj: General analyses for linear models*. [jamovi module]. Retrieved from https://gamlj.github.io/; The jamovi project (2021). *jamovi*. (Version 1.8) [Computer Software]. Retrieved from https://www.jamovi.org.).

## Results

### Number of laboratories participating

Overall, 75 laboratories had enrolled in the scheme and submitted results by the eleventh distribution. While panels were sent to between 45 and 66 laboratories in each distribution, the number submitting results ranged from 37 to 51 over the eleven distributions (Table 2). This number is continually changing—new laboratories enrol after each distribution, laboratories cannot always participate in every distribution for various reasons, and occasionally laboratories have to drop out. The number of samples with results submitted ranged from 324 to 479. The number of laboratories submitting results for lyophilized blood was higher than those submitting DBS results in every distribution.

**Table 2.** Characteristics of EQA results submitted by each distribution

	Distribution										
	1	2	3	4	5	6	7	8	9	10	11
Panels shipped	55	53	56	66	45	51	56	58	59	64	62
Participants submitting results^a^	41	37	45	48	41	49	43	49	43	48	51
# Of laboratories which processed											
Dried blood spots	32	30	36	39	35	42	36	43	39	44	48
Lyophilized blood	40	35	43	47	40	48	42	47	42	46	49
Number of results submitted											
Dried blood spots	159	150	180	195	174	209	185	215	195	220	237
Lyophilized blood	200	174	214	235	199	237	210	235	210	230	242
Total	359	324	394	430	373	446	395	450	405	450	479

^a^Results include samples from labs that submitted results after the EQA electronic submission deadline.

Participating laboratories were located in 42 countries. Twenty-one laboratories (28%) were located in 13 African countries, 17 (23%) were in 11 countries in Asia, and eight laboratories (11%) were in six countries in Europe. Fourteen laboratories (19%) were located in nine countries in South and Central America, 12 (16%) in the two North American countries, and three laboratories (4%) were located in Australia.

All seventy-five laboratories submitted results in at least one distribution. Five laboratories submitted in only one distribution, while eleven submitted in all eleven distributions. However, as the scheme is constantly expanding and new laboratories join at each distribution, some of the laboratories which did not submit results in all distributions did not necessarily miss a distribution, but joined the scheme after it started. For example, of the twelve laboratories which submitted results three or fewer times, seven did not join the scheme until distribution nine or later and so all of these except one had submitted in every distribution since enrolling. Twenty-two laboratories submitted results in all the distributions since they joined the scheme, while by the eleventh distribution, nine laboratories had only missed one distribution since joining the scheme.

### Laboratory profile

All laboratories report being able to detect *P. falciparum*, while eight laboratories detect only *P. falciparum* and no other species. The majority of laboratories (67, 89%) report being able to detect *P. falciparum* and *P. vivax*, with or without other species. *Plasmodium knowlesi* is the least common species detected, with 29 laboratories (39%) able to detect this species, while 57 (76%) and 55 (73%) laboratories report being able to detect *P. ovale* and *P. malariae,* respectively.

### Performance

**Fig. 1:**
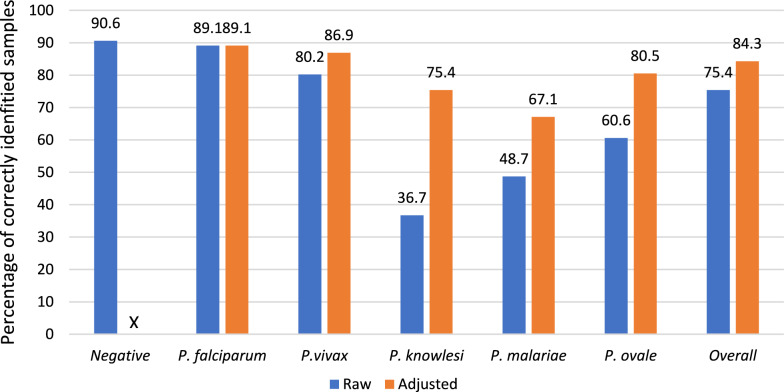
Accuracy of external quality assessment results by species, using raw results and results adjusted by laboratory’s capacity

Across all distributions, laboratories performed best on negative samples, with 90.6% of negative samples being identified correctly (Fig. 1). Among positive samples, *P. falciparum* was the most likely to be identified correctly, at 89.1%. As all laboratories are able to identify *P. falciparum* samples, there was no difference between the raw and adjusted results of these samples. When looking at raw results, without taking into consideration the laboratory profile, 80.2% of *P. vivax* samples were correctly identified, 60.6% of *P. ovale* samples, 48.7% of *P. malariae* samples, and *P. knowlesi* was the most difficult to identify, with only 36.7% identified correctly. However, when results are adjusted according to the laboratory profiles, the percentage of correctly identified *P. knowlesi* samples increased to 75.4%, 67.1% of *P. malariae* samples were correctly identified, as were 86.9% of *P. vivax* samples.

### Referee laboratories

**Fig. 2:**
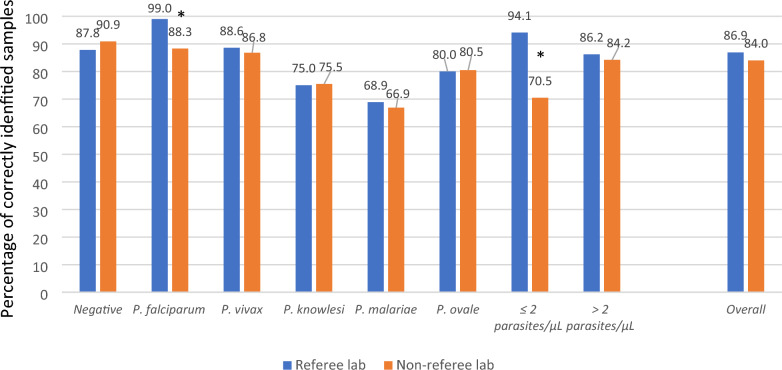
Adjusted accuracy of external quality assessment results for referee vs non referee laboratories according to sample type^a^. *Denotes difference with a p-value below 0.05. ^a^Results adjusted for laboratory capacity

Overall, there was no significant difference in performance between the six referee laboratories that participate in the scheme and non-referee laboratories (86.9% and 84.0%, p = 0.65) (Fig. 2). However, when broken down by species, referee laboratories showed significantly better performance at identifying *P. falciparum* samples (99.0% and 88.3% correctly identified in referee and non-referee laboratories, respectively, p < 0.001). There was no significant difference among any other species, negative samples, or format of sample overall. When broken down by parasite density, referee labs showed markedly better performance than non-referee labs against samples ≤ 2 parasites/µL (94.1% and 70.5%, p = 0.002).

### Performance by sample format and parasite density

**Fig. 3:**
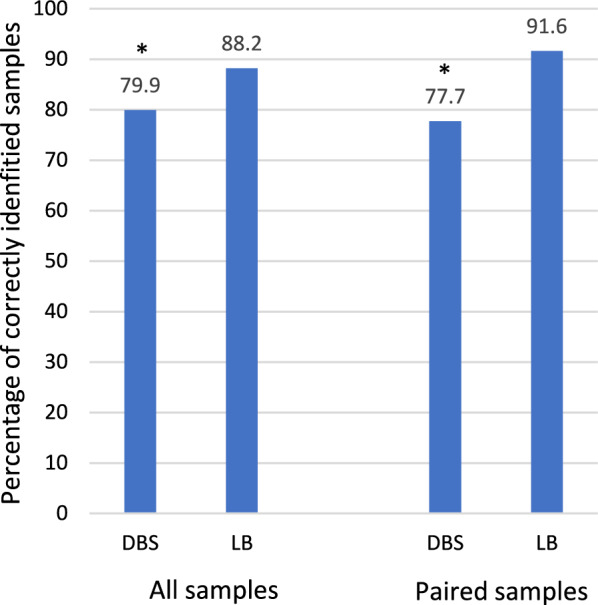
Adjusted accuracy of external quality assessment results for by sample format^a^. *Denotes difference with a p-value below 0.05. DBS: Dried Blood Spots. LB: lyophilized blood. ^a^Results adjusted for laboratory capacity

Performance on analysis of lyophilized blood was more accurate than on DBSs, with 88.2% of lyophilized samples and 79.9% of DBSs correctly identified (p < 0.01) (Fig. 3). Among the paired samples (meaning the samples had identical species and concentrations) included in six of the distributions, accuracy was 91.6% for lyophilized samples and 77.7% for DBSs (p < 0.01).

**Fig. 4.**
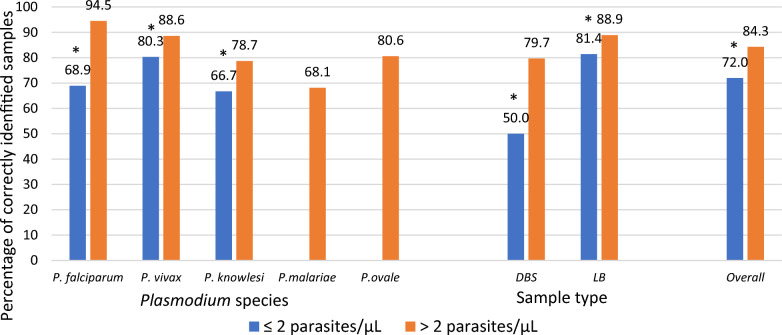
Adjusted accuracy of external quality assessment results by parasite concentration^a^. *Denotes difference with a p-value below 0.05. DBS: Dried Blood Spots. LB: lyophilized blood. ^a^Results adjusted for laboratory capacity. ^b^All samples of *P. malariae* and *P. ovale* were > 2 parasites/µL. Three *P. ovale* sample was pooled and therefore concentration was unknown and not included in the analysis

When samples were broken down into parasite density, higher density samples were better detected overall and on each sample format, where data was available. There was strong evidence to show better performance at densities above 2 parasites/µL, by DBS and lyophilized blood, and among *P. falciparum*, *P. vivax* and *P. knowlesi* samples (Fig. 4). This biggest difference in performance was seen among *P. falciparum* samples, with 68.9% and 94.5% being correctly identified for samples ≤ 2 parasites/µL and > 2 parasites/µL respectively. No comparison was possible for *P. malariae* and *P. ovale*. Difference in performance was especially marked among DBSs below and above 2 parasites/µL, with only 50.0% of DBS samples below 2 parasites/µL being correctly identified compared with 79.7% of those above this threshold (p < 0.01).

### Analysis method used

The most commonly used nucleic acid extraction method, using silica columns, accounted for 60.8% of the samples analysed, and the remaining samples were analysed using seven other methods (Additional file 1). As the majority of labs were using a similar method, it is difficult to draw conclusions about which methods showed the best or worst performance (Additional file 2). The most commonly used amplification method was real-time single target PCR (34.8%), followed closely by nested PCR (33.0%) (Additional file 1). There was no significant difference in performance by method of nucleic acid amplification (Additional file 2).

### Performance of laboratories over time

#### Negative samples

The odds of correctly identifying a parasite negative sample were significantly higher for lyophilized samples compared to DBS (Odds Ratio (OR) 1.618; 95% CI 1.029–2.545; p = 0.037). There was no significant relationship with submission number (p = 0.596), with consistently high odds of correctly detecting negative samples.

#### *Plasmodium falciparum* samples

**Fig 5.**
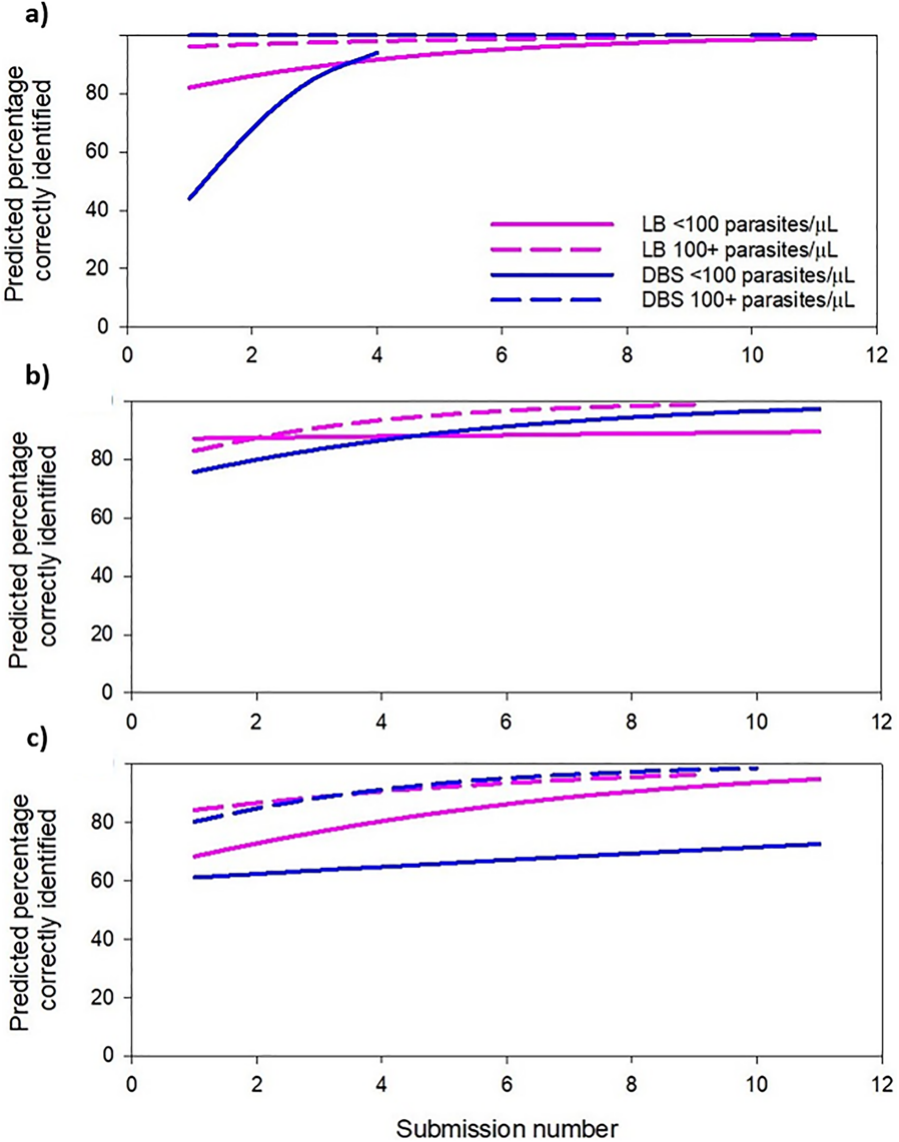
Change in performance by submission number, broken down by Plasmodium species, sample format and concentration for **a** *P. falciparum* samples, **b** *P. vivax* samples, and **c** *P. ovale*, *P. knowlesi* and *P. malariae* samples. DBS: Dried Blood Spots. LB: lyophilized blood

The odds of correctly identifying a sample containing *P. falciparum* parasites were influenced by submission number for samples below 100 parasites/µL, for both DBS and lyophilized blood samples (Fig. 5, Additional file 3). Data were available for four submissions for lower density DBS samples and 11 submissions for higher density samples. For each increase in submission number, the odds of correctly identifying one of these samples below 100 parasites/µL increased by 2.70 for DBS samples (95% CI 1.57–4.65, p < 0.001) and 1.34 for lyophilized blood samples (95% CI 1.15–1.56, p < 0.001). The change in performance was not significant (p > 0.355) for higher density samples, as performance of these samples was consistently high from the beginning of the scheme.

#### *Plasmodium vivax* samples

For each additional submission made by a laboratory, the odds of correctly identifying a *P. vivax* sample increased by 1.28 for lower density DBS samples (95% CI 1.06–1.54, p = 0.010), over 11 submission distributions (Fig. 5, Additional file 4). The change in performance was not significant for lyophilized blood samples of either higher (p = 0.072) or lower density (p = 0.739), and there were insufficient data to model DBS samples above 100 parasites/µL.

### *Plasmodium knowlesi, P. malariae and P. ovale samples*

For samples containing *P. knowlesi, P. malariae* or *P. ovale* the odds of a correct result increased by 1.37 (95% CI 1.12–1.66, p = 0.001) by submission number for DBS samples over 100 parasites/µL, while the change in performance of lower density DBSs was not found to be affected by submission number (p = 0.235) (Fig. 5, Additional file 5). Performance was shown to improve for lower density lyophilized samples, with odds of correctly identifying a sample increasing by 1.24 (95% CI 1.24–1.39, p < 0.001), however while the trend for the higher density lyophilized samples was the same, these results were not statistically significant (p = 0.093).

### Challenges and reasons for non-participation

Despite being enrolled in the scheme for several distributions, some laboratories were unable to participate in every distribution, or had to stop participation. Discussions with laboratory personnel indicated that this inconsistency or discontinuation of the scheme was due to a number of reasons, some of which were outside the control of the laboratory personnel. For example, obtaining import permits in time for importing samples into the country has been problematic for some laboratories since the beginning of the scheme, inability to pay customs fees, availability of reagents, and lack of laboratory staff for the testing can be challenging. The COVID-19 pandemic exacerbated many of these challenges and caused additional struggles for laboratories due to the shutdown of transport routes, laboratory staff time being diverted to other projects and inability of staff to access the laboratories. Departure of the UK from the European Union has led to disruptions for some laboratories located in Europe, as import permits are now required for European laboratories, where they were not required previously.

The majority of laboratories have been able to pay or have had their fees paid by third-parties since the introduction of fees in 2020. On several occasions where a laboratory has been unable to continue due to funding constraints, the WHO has covered the cost of participation. These fees do not cover WHO costs of running the scheme and, therefore, external sources of funds have been mobilized and will continue to be required in the future.

## Discussion

High quality NAAT for malaria diagnosis is essential to support research that informs policy and the development of new medicines, diagnostics and vaccines, as well as strategies to move towards malaria elimination in some settings. Having an EQA scheme like the one presented here can support laboratories to improve their performance and maintain it over time.

Prior to enrolment, laboratories submitted a profile outlining which *Plasmodium* species they could detect to species and genus level. While all laboratories could detect *P. falciparum* and the majority detected *P. vivax*, detection of the other species was much less common, and the performance at detecting these samples was poorer than for *P. falciparum* or *P. vivax*. While *P. falciparum* and *P. vivax* are the two main species infecting people in malaria-endemic countries, it may become increasingly important to detect the less common species using NAATs, as they are less easily detected by RDTs, and high-quality microscopy, which can differentiate between species, is difficult to maintain.

*Plasmodium knowlesi* is now endemic in all Southeast Asian countries except Timor-Leste, and is the most common cause of malaria in some regions [28]. There is no good alternative to NAATs to detect this species as the ability of RDTs to detect *P. knowlesi* is poor [29] and there is no RDT that specifically targets this species or discriminates it from others. Misidentification of *P. knowlesi* as another species by microscopy is due to its similarity to other species (e.g. to *P. malariae,* and to *P. falciparum* at the ring stage) [30]. Therefore, good quality NAATs to detect this species are essential and the lack of a well-performing assay could be a serious hindrance to malaria case management and control in endemic countries.

False-negatives were more common than false-positive results. Some results showed misidentification of species, so while the presence of *Plasmodium* nucleic acids was detected, the correct species was not reported. The consequences of either type of error, or of misidentifying a species, can be critical, not only in clinical practice, but also in certain types of research such as drug efficacy studies.

Performance on samples of lyophilized blood was markedly higher than DBS samples, likely due to the larger volume of blood (500 µL) which can be extracted from lyophilized samples compared to DBS (50 µL), thus increasing the chance of capturing parasite-derived nucleic acids. Comparisons of identical paired samples showed 14 percentage points better accuracy on lyophilized blood than DBSs. The accuracy for DBS samples with parasite densities < 2 parasites/µL was only 50.0% in the scheme. Two parasites/ µL is considered the threshold that NAAT assays should be consistently reaching to offer significant performance advantage over microscopy and RDTs. DBSs are very useful tools for analysing archived samples and are much easier to collect and transport than lyophilized samples, but care must be taken when analysing these types of samples as the limit of detection is higher than for lyophilized samples because the tested sample volume for DBS is usually lower than for lyophilized samples. A study has shown that the concentration of Plasmodium DNA extracted from DBS is much lower than from whole blood, resulting in lower detection of malarial DNA after PCR [31], while another study has shown that when comparable volumes of blood are used in DBS and whole blood format, there was only a moderate reduction in DNA amplification from DBS samples compared to whole blood [32]. Therefore, it must be understood that negative results on DBS samples may not necessarily mean absence of *Plasmodium* parasites, but too few to detect by NAATs on that specimen type.

### Performance over time

The performance of laboratories improved significantly over time, even after adjusting for concentration and sample format, especially for lower density and *P. falciparum* samples. Performance of these samples was generally poorer in the first submissions, and improved rapidly over time participating in the scheme, to become more similar in performance to that on higher density samples. Performance of negative samples and higher density *P. falciparum* samples was very high to begin and has been maintained. As the overall performance has improved, it has started to plateau, so it is now important to help poorer performing laboratories to improve their accuracy, as well as supporting the higher performing laboratories to maintain their performance over time. Establishing a network of laboratories to facilitate higher performing laboratories to support those with lower scores can help to raise the performance of these laboratories. If a laboratory is shown to have poor performance, they can request a repeat panel to be shipped for re-testing, and can be put in contact with an expert who can provide remote technical support, or with other laboratories from the network that might have faced similar challenges and/or are using similar methodologies.

Performance was not shown to be affected by methodology or type of nucleic acid detected. Rather than recommending that laboratories change their methods to another method of extraction, improving performance of the method they are using already is more efficient at improving performance.

While the overall number of laboratories participating in the scheme has increased, the proportion participating in each distribution has remained about the same, with several laboratories in each distribution not submitting results. While some of the factors leading to missed participation in a distribution are outside the control of the lab, it is important to discuss with lab personnel why this is happening, what the main barriers are, and ways in which the coordinators of the scheme can facilitate more consistent participation in the scheme. Factors such as issues with funding or availability of reagents can be problematic. Where laboratories are not able to submit results in time, in exceptional circumstances, the window to submit has been extended. While the scheme was originally free for participants, fees were introduced in distribution seven. Many laboratories are still unable to pay these fees, and need to be encouraged to build the budget for these fees into funding proposals. Several labs did withdrawal participation after introduction of fees.

### Adapting the scheme to track biological threats

The development of such a robust EQA scheme allows for expansion into assessing performance on other aspects of *P. falciparum* infection. Since distribution five, random *P. falciparum* samples have been included which have *pfhrp2* and/or *pfhrp3* gene deletions. These gene deletions have been detected in all malaria endemic regions at varying prevalence and are able to evade detection by the most commonly used (i.e., HRP2-detecting) RDTs [33]. Surveillance efforts to monitor these gene deletions have expanded over the last decade, and their confirmation relies entirely on NAATs. As confirmation of these deletions relies on the absence of a DNA product, it is an especially challenging technique. Including these samples in the panels will allow those laboratories which test for the presence or absence of these genes to assess their ability to identify correctly *pfhrp2* and/or *pfhrp3* gene deletions among *P. falciparum* samples.

An addition to the EQA scheme will be included in future distributions, with *P. falciparum* samples with/without anti-malarial (AM) drug resistance markers, to assess laboratories’ ability to detect a range of currently known drug resistance markers. A pilot phase including *PfK13* single nucleotide polymorphisms, which confer partial resistance to artemisinin drugs, and other partner drug resistance markers is planned for 2025. Laboratories who wish to participate in the AM resistance marker testing scheme will be sent their usual panel of samples for EQA testing, along with an additional panel with *P. falciparum* samples with/ without AM resistance markers. Adding this new scheme onto the existing EQA scheme saves on logistic and shipping costs and benefits both the participating laboratories and the coordinators of the scheme.

Inclusion of paired samples will also allow laboratories to assess their performance of molecular correction, an important tool used to differentiate recrudescence with new infections for interpreting therapeutic efficacy study outcomes.

### Future role of NAAT

The COVID-19 pandemic highlighted the role that NAAT, backed up by EQA schemes, can play in infectious diseases diagnostics [34]. The pandemic led to a great expansion in NAAT capacity in many countries [35, 36] and countries want to maintain and leverage this enhanced capacity for expansion of molecular diagnostics to other diseases. It is hoped that this will then lead to an increase in demand for the malaria molecular EQA scheme. The results from the first eleven distributions of this EQA scheme indicate that performance of laboratories has increased over time, showing the positive impact that EQA schemes can have on NAAT performance.

The scheme coordinators are aware that there are a large number of laboratories that perform malaria NAATs that are not yet participating in the scheme, so an ongoing activity is to determine how best to encourage them to join the scheme. Another malaria molecular scheme is run by UK NEQAS, using LB samples only and laboratories can decide which is more suitable for them, depending on their needs.

These results show that the goal of this EQA scheme has been achieved in that it appears to have facilitated improved NAAT performance of laboratories over time. It has also revealed areas where improvement is still needed, including detection of very low-density infections (< 2 parasites/µL) and minor species. It is also proving itself to be a strong backbone to efficiently expand to meet needs for monitoring proficiency at detecting emerging biological threats such as *pfhrp2/3* deletions and anti-malarial drug resistance. Donors should make participation in an EQA scheme a requirement for laboratories they are financing to apply NAAT for surveillance or research purposes, and countries can take the initiative to embed such schemes into their own national assessment programmes, rather than the current system of vertical schemes. This will lead to improved performance and accountability of those laboratories that conduct NAATs to inform surveillance, disease management and research.

## Supplementary Information

Additional file 1.

Additional file 2.

Additional file 3.

Additional file 4.

Additional file 5.

## Data Availability

No datasets were generated or analysed during the current study.
