# Supplementary material for: WHO malaria nucleic acid amplification test external quality assessment scheme: results of eleven distributions over 6 years
Source: Malar J. 2025 Mar 23;24:94. doi: 10.1186/s12936-025-05282-0 (PMC11929988; doi:10.1186/s12936-025-05282-0)
Supplement: Supplementary file 1 — Additional file 1. [file 12936_2025_5282_MOESM1_ESM.docx]

Additional file 1. Characteristics of extraction (**a**) and amplification (**b**) methods used by participating laboratories across eleven distributions. Percentages of samples using each methodology are shown. Methods of extraction and amplification included in ‘other’ category were not described by participants.
