# Supplementary material for: WHO malaria nucleic acid amplification test external quality assessment scheme: results of eleven distributions over 6 years
Source: Malar J. 2025 Mar 23;24:94. doi: 10.1186/s12936-025-05282-0 (PMC11929988; doi:10.1186/s12936-025-05282-0)
Supplement: Supplementary file 2 — Additional file 2. [file 12936_2025_5282_MOESM2_ESM.docx]

Additional file 2.

Percentage of samples correctly identified by extraction and amplification method, adjusted for labototory’s capacity to detect species

| Method | Number of samples analysed using this method | Percentage of samples correctly identified |
| --- | --- | --- |
| Extraction method | | |
| Qiagen: Silica column | 2,738 | 85.5 |
| BioRad: Chelex100 | 335 | 82.2 |
| NucliSENS easy MAG | 75 | 85.3 |
| Boom guanidine silica extraction | 73 | 97.3 |
| Qiagen: QIA symphony | 68 | 94.2 |
| MagnaPur | 52 | 96.2 |
| BioRad: InstaGene | 15 | 40.0 |
| Other | 896 | 80.4 |
| Not reported | 253 | 79.8 |
| Amplification method | | |
| Real time single target | 1,567 | 83.7 |
| PCR: Nested | 1,485 | 82.8 |
| PCR: Multiplex | 926 | 84.6 |
| PCR: Single target | 87 | 87.4 |
| LAMP | 25 | 100 |
| Other | 296 | 90.5 |
| Not reported | 119 | 87.4 |
