# Supplementary material for: WHO malaria nucleic acid amplification test external quality assessment scheme: results of eleven distributions over 6 years
Source: Malar J. 2025 Mar 23;24:94. doi: 10.1186/s12936-025-05282-0 (PMC11929988; doi:10.1186/s12936-025-05282-0)
Supplement: Supplementary file 3 — Additional file 3. [file 12936_2025_5282_MOESM3_ESM.docx]

Additional file 3. Odds ratios and significance of submission number as a predictor of correct *P. falciparum* sample identification

| Sample type | Density group | Submission no. included in model | No. observations (No. labs) | % Correct | OR (95% CI) | P-value | R_c_^2^ |
| --- | --- | --- | --- | --- | --- | --- | --- |
| DBS | <100 | 1-4 | 135 (51) | 54.1 | 2.70 (1.57 – 4.65) | **<0.001** | 0.439 |
|  | 100+ | 1-11 | 402 (66) | 95.0 | 1.15 (0.79 – 1.66) | 0.468 | 0.965 |
| Lyophilized Blood | <100 | 1-11 | 395 (71) | 88.4 | 1.34 (1.15 – 1.56) | **<0.001** | 0.372 |
|  | 100+ | 1-8 | 299 (63) | 98.0 | 1.25 (0.78 – 1.99) | 0.355 | 0.048 |

R_c_^2^ is the conditional pseudo-R2 which is the variance explained by the fixed and random effects together over the total (expected) variance of the dependent variable.
