# Supplementary material for: WHO malaria nucleic acid amplification test external quality assessment scheme: results of eleven distributions over 6 years
Source: Malar J. 2025 Mar 23;24:94. doi: 10.1186/s12936-025-05282-0 (PMC11929988; doi:10.1186/s12936-025-05282-0)
Supplement: Supplementary file 4 — Additional file 4. [file 12936_2025_5282_MOESM4_ESM.docx]

Additional file 4. Odds ratios and significance of submission number as a predictor of correct *P. vivax* sample identification

| Sample type | Density group | Submission no. included in model | No. observations (No. labs) | % Correct | OR (95% CI) | P-value | R_c_^2^ |
| --- | --- | --- | --- | --- | --- | --- | --- |
| DBS | <100 | 1-11 | 288 (61) | 84.7 | 1.28 (1.06 – 1.54) | **0.010** | 0.382 |
|  | 100+ | 1-3 | 36 (36) | 88.9 | Not modelled due to insufficient data | | |
| Lyophilized Blood | <100 | 1-11 | 341 (73) | 85.0 | 1.02 (0.89 – 1.18) | 0.739 | 0.229 |
|  | 100+ | 1-9 | 131 (52) | 96.2 | 1.44 (0.97 – 2.14) | 0.072 | 0.143 |

R_c_^2^ is the conditional pseudo-R2 which is the variance explained by the fixed and random effects together over the total (expected) variance of the dependent variable.
