# Supplementary material for: WHO malaria nucleic acid amplification test external quality assessment scheme: results of eleven distributions over 6 years
Source: Malar J. 2025 Mar 23;24:94. doi: 10.1186/s12936-025-05282-0 (PMC11929988; doi:10.1186/s12936-025-05282-0)
Supplement: Supplementary file 5 — Additional file 5. [file 12936_2025_5282_MOESM5_ESM.docx]

Additional 5. Odds ratios and significance of submission number as a predictor of correct *P. knowlesi*, *P. malariae* and *P. ovale* sample identification

| Sample type | Density group | Submission no. included in model | No. observations (No. labs) | % Correct | OR (95% CI) | P-value | R_c_^2^ |
| --- | --- | --- | --- | --- | --- | --- | --- |
| DBS | <100 | 1-11 | 410 (65) | 63.4 | 1.05 (0.97 – 1.15) | 0.235 | 0.166 |
|  | 100+ | 1-10 | 277 (63) | 71.1 | 1.37 (1.12 – 1.66) | **0.001** | 0.777 |
| Lyophilized Blood | <100 | 1-11 | 340 (70) | 77.1 | 1.24 (1.11 – 1.39) | **<0.001** | 0.349 |
|  | 100+ | 1-9 | 273 (65) | 86.1 | 1.22 (0.97 – 1.54) | 0.093 | 0.316 |

R_c_^2^ is the conditional pseudo-R2 which is the variance explained by the fixed and random effects together over the total (expected) variance of the dependent variable.
